# Simultaneous Total Knee Arthroplasty and Ankle Arthrodesis for Charcot Neuroarthropathy

**DOI:** 10.1155/2019/6136409

**Published:** 2019-12-07

**Authors:** Koji Nozaka, Naohisa Miyakoshi, Yusuke Yuasa, Motoki Mita, Yoichi Shimada

**Affiliations:** Department of Orthopedic Surgery, Akita University Graduate School of Medicine, Hondo, Akita 010-8543, Japan

## Abstract

**Introduction:**

Charcot neuroarthropathy is a progressive, deforming pathology of the bone and joints, especially affecting the knees and ankles. Although it is rare, it leads to considerable morbidity. The treatment of Charcot arthropathy of the knee and ankle remains controversial. Many authors suggest that knee involvement is an absolute contraindication to total knee arthroplasty. In recent years, however, several studies have shown satisfactory results for total knee arthroplasty. In the ankle, external fixators have recently been advocated by many authors. Their main advantages are that they permit monitoring of soft tissue healing and avoidance of more invasive surgery. Simultaneous Charcot knee and ankle joint surgery involving total knee arthroplasty (TKA) and ankle arthrodesis is rare and challenging and can lead to major complications if not addressed appropriately.

**Case Presentation:**

The case of a 71-year-old woman who underwent simultaneous total knee arthroplasty and ankle arthrodesis for severe neurosyphilitic Charcot arthropathy (Eichenholtz classification stage III) and was evaluated three years after surgery is reported. Deformities of the left knee joint and ankle developed. The left leg was shorter by 20 mm, with a functional leg length discrepancy. The patient was limping, and marked varus instability of the left ankle was observed during the stance phase of walking. Postoperatively, the patient was able to walk without assistance, confirming improvement of mobility.

**Conclusion:**

To the best of our knowledge, this is the first report of combined, simultaneous neurosyphilitic Charcot knee and ankle joint surgery involving TKA and ankle arthrodesis. It was an effective surgical method that maintained leg length and achieved satisfactory alignment without an autologous iliac bone graft.

## 1. Introduction

Simultaneous Charcot knee and ankle joint surgery involving total knee arthroplasty (TKA) and ankle arthrodesis is rare and challenging and can lead to major complications if not approached appropriately [1, 2]. Neuropathic arthropathy, such as a Charcot joint, is characterized by rapidly progressive bone destruction in the setting of impaired nociceptive and proprioceptive innervation to the involved joint [3]. The surgical options for treatment of Charcot neuroarthropathy remain poorly defined, particularly for the knee and ankle joints [4]. Neuropathic arthropathy can be associated with tabes dorsalis, a unique manifestation of late, tertiary neurosyphilis that may arise in individuals with untreated syphilis [3]. Historically, Charcot joints were most commonly associated with syphilitic (*Treponema pallidum*) infections before the introduction of effective antibiotics in the mid-20th century. Jean-Marie Charcot's description of neuropathic joints in 1868 described patients with tabes dorsalis, a form of tertiary neurosyphilis that may develop months to decades after the patient's initial infection [5]. The technical challenges encountered during TKA and ankle fusion in patients with neuropathic arthropathy, particularly in those with significant deformities, may require a high degree of surgical skill and experience [6, 7]. These conditions are thought to be responsible for Charcot joint disease, which is characterized by the development of bone destruction. The management of patients with Charcot joint includes early detection of disease, protection of further injury to cartilage, and prevention of disease progression. However, the degenerative process with gross deformity and destruction may lead to significant disability in some cases. Therefore, a case of simultaneous surgery involving TKA and ankle arthrodesis for severe neurosyphilitic Charcot arthropathy is described.

## 2. Case Presentation

**Figure 1:**
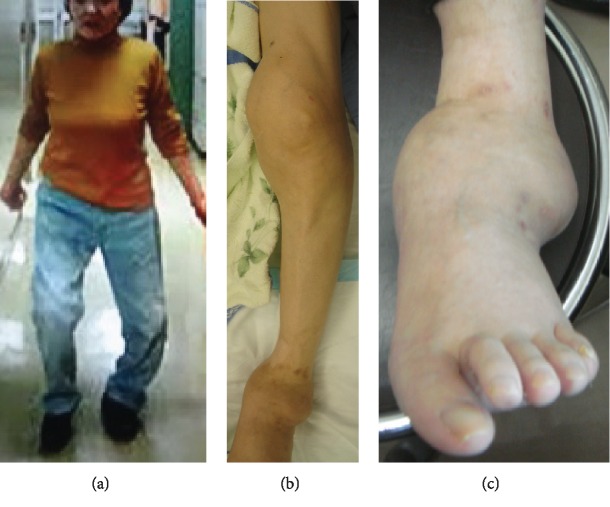
Clinical picture. (a) The patient is limping, but not using any assistive device. Marked varus instability of the left ankle is observed during the stance phase of walking. (b) There is marked swelling but no instability of the left knee joint. (c) The left ankle joint has marked swelling, and the foot is medially dislocated. A skin ulcer has formed at the lateral malleolus.

This case involved a 71-year-old woman with a primary complaint of gait disturbance. The patient had a history of hypertension and cerebral infarction, and she had been taking oral antihypertensive and antiplatelet agents. The patient had no sequelae of cerebral infarction, after which she had a good course taking oral antihypertensive and antiplatelet agents. Diabetes mellitus is a common cause of Charcot arthropathy, but the patient did not have diabetes mellitus. The patient also had no family history of a cause of Charcot arthropathy or a neurological disease. She had a history of the present deformities of the left knee joint and ankle from around 65 years of age, but the patient left them untreated because she did not have severe pain. From around 69 years of age, varus deformity of the left ankle progressed. The patient developed difficulty walking and, therefore, visited our department for consultation. Physical examination showed that the left leg was shorter by 20 mm, with a functional leg length discrepancy. The patient was limping, but not using any assistive device. Marked varus instability of the left ankle was observed during the stance phase of walking (Figure 1). Range of motion in the left knee joint was extension of -15° and flexion of 75°. There was marked swelling, but no instability (Figure 1). The left ankle joint had marked swelling, and the foot was medially dislocated. A skin ulcer had formed at the lateral malleolus (Figure 1).

### 2.1. Imaging Findings

**Figure 2:**
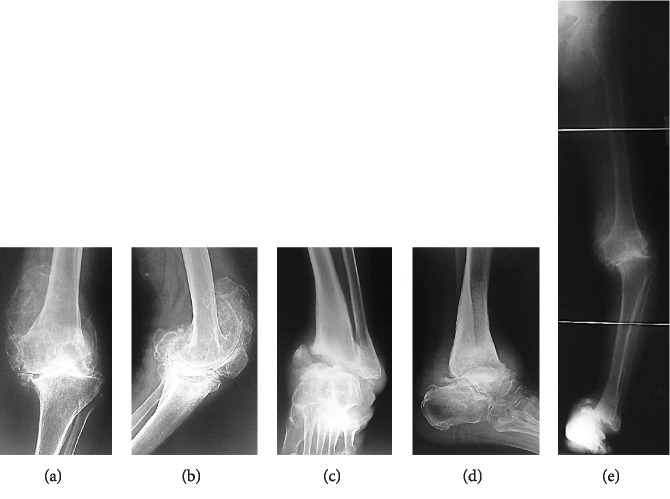
Preoperative radiographs of the knee and ankle. (a, b) Marked narrowing at the joint space, depression and an osseous defect of the medial tibial articular surface, and marked proliferative changes of the femoral condyle in the left knee. (c, d) Medial dislocation of the talus bone, accompanied by an old fracture of the medial malleolus, an osseous defect of the distal medial tibial articular surface, and an osseous defect of the proximal talus articular surface in the left ankle joint. (e) Lower extremity full-length standing X-ray shows a femorotibial angle (FTA) of 202°.

Plain X-ray of the left knee revealed marked narrowing at the joint space, depression and an osseous defect of the medial tibial articular surface, and marked proliferative changes of the femoral condyle (Figure 2). Plain X-ray of the left ankle joint showed medial dislocation of the talus bone, accompanied by an old fracture of the medial malleolus, an osseous defect of the distal medial tibial articular surface, and an osseous defect of the proximal talus articular surface (Figure 2). A lower extremity full-length standing X-ray showed a femorotibial angle (FTA) of 202° (Figure 2).

The patient had an HbA1c level of 6.1% and a fasting glucose level of 103 mg/dL; the patient was not considered to have diabetes. Syringomyelia was not observed on whole-spine MRI. The *Treponema pallidum* hemagglutination test was positive. White blood cell and C-reactive protein levels were normal. There were no general bacteria on culture of synovial fluid from the knee and ankle joints. On electrophysiological testing with the nerve conduction velocity test, a bilateral delay in motor nerve conduction velocity was observed in the deep peroneal nerve, and similarly, a bilateral delay of sensory nerve conduction velocity was observed in the sural nerve.

### 2.2. Surgical Technique

**Figure 3:**
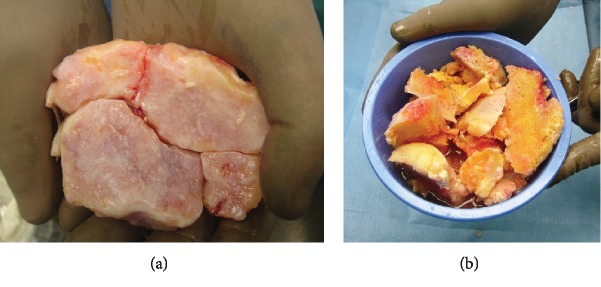
Intraoperative picture. (a) Giant loose bodies surrounding the femur. (b) The large mass of local bone is saved for ankle arthrodesis.

Total knee replacement and ankle (tibiotalar arthrodesis) fusion were performed with the patient under general anesthesia in the supine position and a thigh tourniquet. The medial parapatellar approach was used for the knee joint. A marked intra-articular osteophyte and formation of giant loose bodies surrounding the femur were observed (Figure 3). The large mass of local bone was saved for ankle arthrodesis (Figure 3).

**Figure 4:**
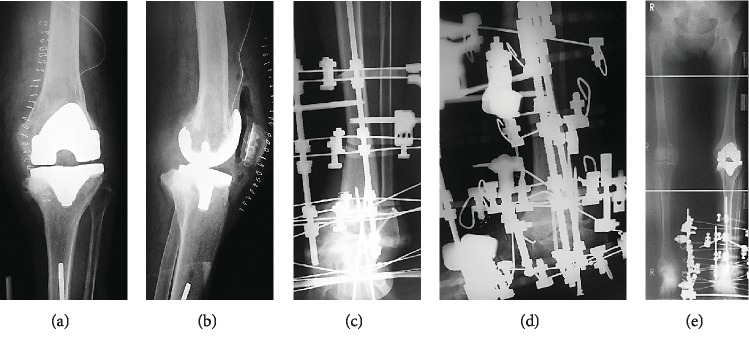
Postoperative radiographs of the knee and ankle. (a, b) A 5 mm defect is observed at the medial tibia. After drilling, cement is used as filling. The posterior-stabilized (PS) type is fixed with cement. (c–e) Ilizarov external fixation is performed on the ankle joint. The bone is repositioned to a neutral position without shortening.

A 5 mm defect was observed at the medial tibia. After drilling, cement was inserted as filling. The posterior-stabilized (PS) type was fixed with cement (Figure 4), and the wound was closed. Next, surgery on the ankle joint was performed using an anterior approach. The distal tibial articular surface and proximal talus articular surface were rasped, drilling was performed, and the bone was repositioned to a neutral position without shortening (Figure 4). Subsequently, an autologous bone graft from the knee was placed at the talocrural joint space, and the wound was closed. Ilizarov external fixation was used to fix the bone. First, two straight wires and two half pins were inserted into the proximal-to-mid tibia and attached to the proximal full ring, and then, six straight wires were inserted into the calcaneus and attached to the distal foot ring. In addition, six straight wires were inserted into the distal tibia for strong fixation (Figure 4).

### 2.3. Course

The total operative time for the combined procedure was 152 minutes. The hemoglobin concentration decreased from 13.9 mg/dL preoperative1y to 9.8 mg/dL on postoperative day 1, but the patient was not transfused. On postoperative day 3, knee drainage material was removed, oral antiplatelet administration was resumed, and walking with full weight-bearing was permitted. Hospitalization was prolonged for 14 days. The ankle fusion appeared to have healed by 9 weeks, as evidenced radiographically and on CT by the absence of a lucent line at the arthrodesis site. The external fixator of the ankle was removed on postoperative day 92. At follow-up 7 years after the simultaneous surgery, the patient was satisfied with the procedure. Her American Orthopaedic Foot & Ankle Society scale ankle/hindfoot scale scores improved, from 32 before surgery to 91 after surgery. SF36 scores improved, from 24.1 (physical component summary) before surgery to 51.1 (physical component summary) after surgery and from 16.5 (mental component summary) before surgery to 59.5 (mental component summary) after surgery. The Visual Analog Scale improved, from 75 before surgery to 0 after surgery. The Knee Society subjective and physical examination score improved, from 25 before surgery to 85 after surgery. The patient's clinical score was checked on the day before surgery and 3 months after Ilizarov external fixation removal.

## 3. Discussion

**Figure 5:**
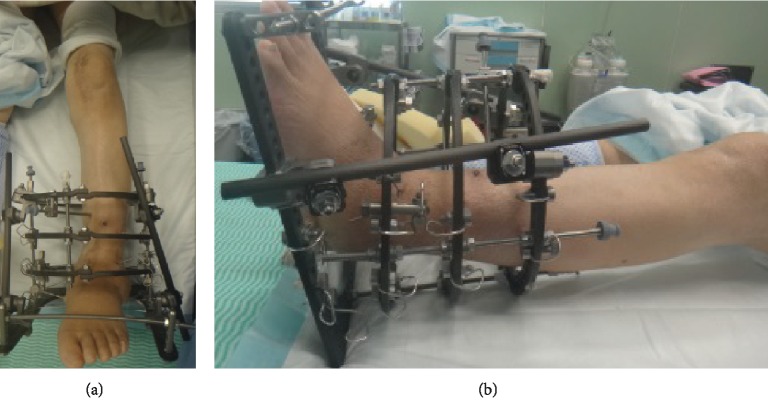
Clinical picture. (a, b) Ilizarov external fixation as a method of Charcot ankle arthrodesis.

This case report highlights a 71-year-old woman with simultaneous Charcot joint surgery involving TKA and ankle arthrodesis. Charcot neuroarthropathy is a rare, progressive, deforming disease of bone and joints, especially affecting the knee and ankle and leading to considerable morbidity. To the best of our knowledge, this is the first report showing the combination of simultaneous neurosyphilitic Charcot knee and ankle joint surgery involving TKA and ankle arthrodesis. The combination of simultaneous TKA and ankle arthrodesis for Charcot knee and ankle joint surgery is rare and challenging, and it can lead to major complications if not approached appropriately. Recently, there has been an increasing number of reports using Ilizarov external fixation as a method of Charcot ankle arthrodesis (Figure 5).

Such an increase was triggered by the widespread usage and the technological advancement of the management of the Ilizarov external fixator, which is known to have a rigid fixation force [8–11]. Ankle arthrodesis has been established as a reasonable salvage procedure for many patients with advanced ankle degeneration and medical comorbidities [12]. Patients with Charcot neuroarthropathy have compromised bone healing, and they have traditionally been treated nonoperatively or with amputation. The Ilizarov method has been used in these complex cases [3, 8, 9].

A uniform consensus has not been obtained in terms of which joint to treat first when there is simultaneous impairment and misalignment of the knee and ankle joints on one side of the body [13]. In the present case, marked varus instability was observed at the ankle, and a skin ulcer had already formed at the lateral malleolus; thus, there was no question that treatment of the ankle had to be considered first [14]. However, if the ankle joint alone was treated first and the knee joint was subsequently treated at the second stage, varus alignment of the overall lower extremities would not have improved. Therefore, with the increased load stress to the medial ankle, it may have been disadvantageous for the bone union of the talocrural joint in ankle arthrodesis. Particularly in Charcot joints, poor alignment causes early destruction of the adjacent joint, and thus, realignment of the overall lower extremities becomes important. For this reason, it was decided to treat both knee and ankle joints in one stage in the present case, and as a result, there has been no loosening of the artificial joint 7 years after surgery.

The surgical treatment of Charcot joint of the knee remains controversial. Previously, arthrodesis that provides support was common at the time of treatment for Charcot knee joint. However, knee arthrodesis is known to markedly reduce patients' quality of life. In particular, the lifestyle in Japan is different from that in Western countries, and there are many occasions where a sitting position is required. Thus, patient satisfaction with knee arthrodesis is low. In recent years, depending on the patient, TKA is considered as one of the treatment options, and satisfactory outcomes have been reported. Nonetheless, the long-term outcome is not as stable as for other joint diseases; therefore, careful adaptation, postoperative lifestyle guidance, and explanation of the characteristics and pathology of the disease to the patients themselves to obtain their understanding are thought to be necessary.

**Figure 6:**
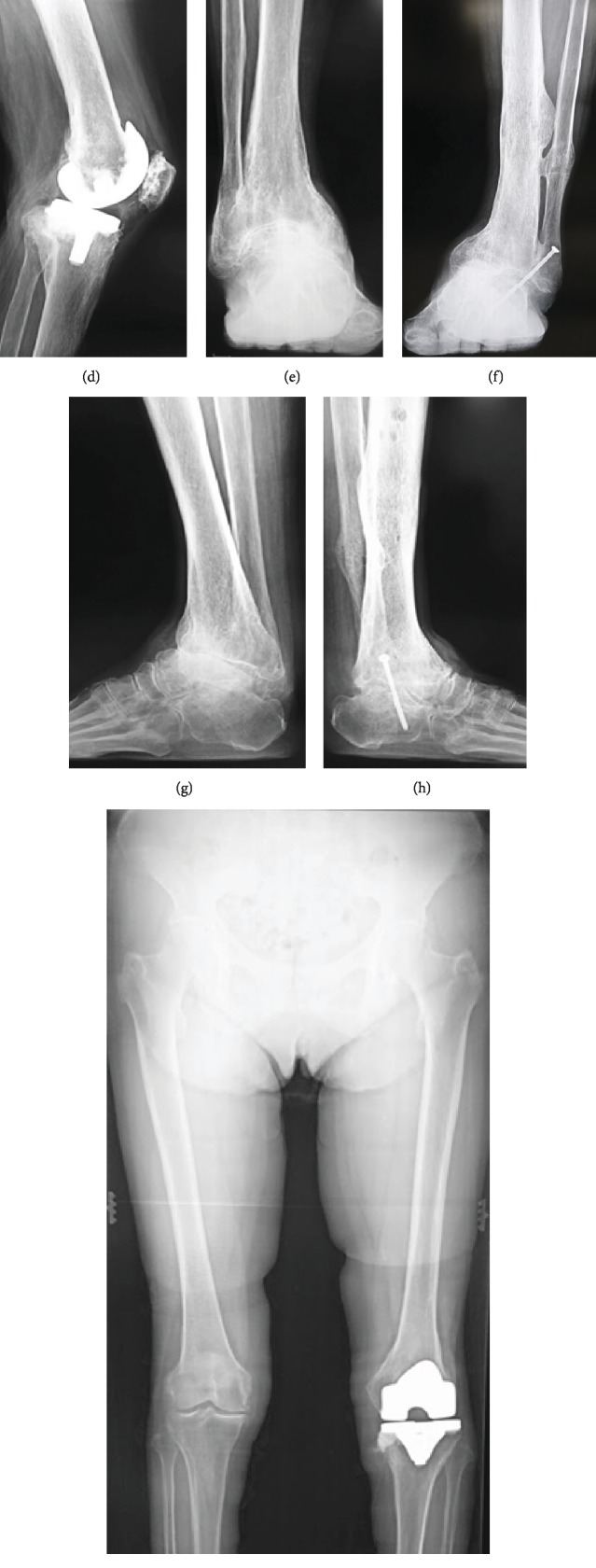
X-rays seven years after surgery. (a–i) It was possible to avoid extreme shortening and to obtain satisfactory alignment with the same leg length as the unaffected side.

The mean length of hospital stay after TKA in Japan is approximately 30 days, and the mean length of hospital stay after ankle arthrodesis is approximately 45 days. In the present case, the patient made satisfactory progress to the point that discharge was possible at 14 days after surgery. In the present case, by using resected bone and large amounts of loose bodies that were generated from TKA in arthrodesis of the ankle with a massive osseous defect, it was possible to avoid extreme shortening and to obtain satisfactory alignment with the same leg length as the unaffected side (Figure 6) [15].

We consider this to be the greatest advantage of performing simultaneous surgery in this case. The present patient did not have instability in the knee joint, but rather had severe contracture. Also, after a cerebral infarction, the patient did not exercise at all and was spending daily life with low activity. Therefore, TKA was performed using the usual posterior-stabilized (PS) type instead of using implants with a stem that are highly restrictive. For the knee, no unnecessary detachment procedures were performed, and it was decided to concentrate on performing TKA with great support and satisfactory alignment. As a result, the operative time and tourniquet time were shortened. This is thought to be a significant factor related to why the present patient had a very good clinical course without complications such as skin problems or thrombosis. In the future, careful follow-up over a long period of time will be necessary. Nonetheless, simultaneous surgery of the knee and ankle joints to treat Charcot joints is an effective surgical method that maintains leg length and achieves satisfactory alignment without an autologous iliac bone graft.

## 4. Conclusions

To the best of our knowledge, this is the first report showing the combination of simultaneous neurosyphilitic Charcot knee and ankle joint surgery involving TKA and ankle arthrodesis. It was an effective surgical method that maintained leg length and achieved satisfactory alignment without an autologous iliac bone graft.

## Abbreviations

TKA: Total knee arthroplasty.
